# Warmblood fragile foal syndrome type 1 mutation (*PLOD1* c.2032G>A) is not associated with catastrophic breakdown and has a low allele frequency in the Thoroughbred breed

**DOI:** 10.1111/evj.13182

**Published:** 2019-10-04

**Authors:** R. R. Bellone, N. R. Ocampo, S. S. Hughes, V. Le, R. Arthur, C. J. Finno, M. C. T. Penedo

**Affiliations:** ^1^ Veterinary Genetics Laboratory School of Veterinary Medicine University of California‐Davis Davis California USA; ^2^ Department of Population Health and Reproduction School of Veterinary Medicine University of California‐Davis Davis California USA; ^3^ School of Veterinary Medicine University of California‐Davis Davis California USA

**Keywords:** horse, catastrophic breakdown, warmblood fragile foal syndrome, WFFS procollagen lysine 2‐oxoglutarate 5‐dioxygenase1

## Abstract

**Background:**

Catastrophic fractures are among the most common cause of fatalities in racehorses. Several factors, including genetics, likely contribute to increased risk for fatal injuries. A variant in the procollagen‐lysine, 2‐oxoglutarate 5‐dioxygenase1 gene (PLOD1 c.2032G>A) was shown to cause Warmblood fragile foal syndrome type 1 (WFFS), a fatal recessive defect of the connective tissue. Screening of multiple horse breeds identified the presence of the WFFS allele in the Thoroughbred. PLOD1 is involved in cross‐linking of collagen fibrils and thus could potentially increase the risk of catastrophic breakdown.

**Objectives:**

Estimate the frequency of the WFFS allele (*PLOD1* c.2032G>A) and determine if it is a risk factor for catastrophic breakdown in the Thoroughbred.

**Study design:**

Case–control genetic study.

**Methods:**

Genomic DNA from hair and/or tissue samples was genotyped for the WFFS allele. Fisher’s Exact tests were performed to compare allele and carrier frequencies between the case cohort (catastrophic breakdown, n = 22) and several cohorts with no record of injury (n = 138 raced/trained at same track and season and n = 185 older than 7 years and raced during same season), nonracers (n = 92), and a random sample without consideration for racing history (n = 279).

**Results:**

The frequency of the PLOD1 c.2032G>A variant in the Thoroughbred breed is low (1.2%). Seventeen of 716 Thoroughbreds tested were carriers (2.4%) and no WFFS homozygotes were detected. Only one catastrophic breakdown case carried the WFFS allele. No statistically significant difference in allele or carrier frequency was identified between case and control cohorts (P>0.05 in all comparisons performed).

**Main limitations:**

This study evaluated cases from one single track.

**Conclusions:**

This study demonstrated that the PLOD1 c.2032G>A associated with WFFS is present at very low frequency in Thoroughbreds and is not a genetic risk factor for catastrophic breakdown.

## Introduction

The Thoroughbred horse is selectively bred for racing performance, among other traits. Racing, like any other sport, has inherent risk of injuries. Catastrophic fractures, resulting from significant bone or soft tissue injury, carry a poor clinical prognosis and are the most common cause of euthanasia or spontaneous death in racehorses 1, 2. Investigating equine catastrophic musculoskeletal injury over a span of 27 years from 21 published studies showed a pooled incidence of 1.17 (0.90–1.44, 95% CI) injuries per 1000 starts 3. The Equine Injury Database, which tracks the frequency, types and outcome of racing injuries in the United States across 118 tracks since 2008, recently reported fatality rates for 2018 to be 1.68 per 1000 starts (http://jockeyclub.com/default.asp?section=Advocacy%26area=10).

Given the critical nature of these injuries, numerous epidemiological studies have focused on identifying key risk factors for racehorse fatality, with close to 300 different factors investigated. A recent meta‐analysis comparing 65 studies from 1990 to 2017 identified several horse‐specific (age, sex, and race class), race‐specific (track conditions, race distance, and field size) and management‐specific (time since previous start, number of starts, and previous injury, among others) factors that demonstrate consistent evidence for risk of catastrophic injury at repeatable sites due to cycle loading of the limbs in high speed work 3. Data from several studies support pre‐existing lesions at the distal end of metacarpal three or metatarsal three as a predisposing risk factor for catastrophic fetlock injuries 4, 5, 6, 7, 8, 9, 10. Cumulative damage of the articular cartilage and subchondral bone, resulting from repetitive strain, is believed to lead to risk for impaired bone remodelling and skeletal failure 1, 11, 12.

Susceptibility to catastrophic fracture may be due to differences among horses in the rate at which bone remodelling occurs which is hypothesised to have a genetic basis 13. The heritability of distal limb fracture was reported to range from 0.21 to 0.37 14, supporting an inherited component and justification for further studies to identify associated or causal variants that enable marker‐assisted selection and identification of susceptible horses that may benefit from an altered training and racing regimens. A genome‐wide association study identified loci on ECA18 and ECA1 associated with distal limb fracture 13. Significant associations with fracture risk were also reported for loci on ECA8, 22, and 31, based on the REML analysis. This same study identified several positional candidate genes involved in bone development, however; to date no causal variants have been reported 13. Therefore, the precise role that genetic variants play in bone metabolism and susceptibility to catastrophic fracture remains to be investigated.

Several genetic variants identified in the horse have been linked to connective tissue disorders and these may be important contributors to catastrophic breakdown. The aims of the current study were to estimate the allele frequency of one of these variants, namely PLOD1 c.2032G>A p.Gly678Arg, in the Thoroughbred population and to determine if allele or carrier frequency differed between catastrophic breakdown case and control cohorts. PLOD1 c.2032G>A has been reported to cause Warmblood fragile foal syndrome type 1 (WFFS) 15. This syndrome has been characterised in Warmblood breeds and presents as hyperextensible, abnormally thin and fragile skin that results in open lesions, as well as, lax and hyperextensible limb joints 15, 16, 17, 18. Important for collagen biosynthesis, PLOD1 catalyses the hydroxylation of lysine residues in Xaa‐Lys‐Gly‐collagen sequences stabilising intermolecular collagen crosslinks and providing strength and support to many body tissues 19. Screening of multiple horse breeds during the commercialisation of this test at the Veterinary Genetics Laboratory, University of California, Davis, USA (VGL) identified the presence of the WFFS allele in the Thoroughbred breed. The presence of this allele in the Thoroughbred, together with the established role of PLOD1 in proper collagen cross‐linking, led to speculation that the *PLOD1* c.2032G>A allele could be associated with catastrophic breakdown in Thoroughbred horses. In this study, we aimed to determine if the *PLOD1* c.2032G>A allele frequency differs between cohorts of Thoroughbred horses known to have catastrophic injuries during a race or in training compared with those raced with no record of injury, and those not raced.

## Materials and methods

### Samples

#### Case cohort

Twenty‐two catastrophic breakdown fatalities were identified for inclusion in this study (Table 1). To minimise other potential variables that contribute to catastrophic fracture, cases that occurred during the racing season (12/2018–3/2019) from a single track were included. Thirteen horses were injured during a race and nine were injured during training. Characterisation of injury type is described in Table 2. Genomic DNA was obtained from either hair (n = 12) banked at the VGL or kidney tissue obtained from banked samples submitted through the California Animal Health and Food Safety Laboratory System following the protocols of California State Racing Board (n = 2) or both hair and kidney tissue (n = 8). DNA isolation from hair was done as previously described 20 and from kidney samples using 3 mm of tissue and an alkaline lysis protocol as described in 21. To confirm sample ID, DNA samples were PCR‐amplified for horse identification markers using the commercially available genetic marker test offered at the VGL. All 22 case samples had genetic profiles on file at the VGL. Sample and horse identification was confirmed by comparing genetic profiles from the DNA extracted for this study to those profiles from previous testing performed as part of the parentage‐testing program for The Jockey Club. Additionally, for the eight samples with both hair and kidney tissue available, genetic profiles were compared between the DNA extracted from the two tissue types and to the profile in the database. All samples matched the expected genetic profile.

**Table 1:** Case and control cohort information. Presented are sample size, number of males and females in each cohort, average age of the animals and age range for the cohort

Cohort	Sample size	Males	Females	Average age	Age range
Case cohort: catastrophic breakdown	22	14	8	4.05	3–7
Control cohort A: same track and same season as cases	138	79	59	4.23	3–8
Control cohort B: horses >7 years old and raced during same season as cases	185	132	53	7.62	7–9
Control cohort C: non‐racers	92	40	52	7.06	5–9
Control cohort D: random population	279	138	141	6.76	1–27

**Table 2:** Catastrophic fracture case cohort. The distribution of cases and bone or joint involved across all four limbs is summarised

Injured limb	Number of cases	Bone or joint involved
Left forelimb	6	Fetlock (n = 5); Carpus (n = 1)
Right forelimb	11	Fetlock (n = 11)
Left hindlimb	3	Metatarsal III (n = 3)
Right hindlimb	2	Metatarsal III (n = 2)

#### Control cohorts

Genomic DNA was isolated from banked hair follicles, as described above 20, from several control cohorts that included A) horses that race or trained at the same track and during the same racing season as the case cohort but with no record of injury (n = 138), B) horses 7 years of age or older that raced during the same racing season, at any US racetrack, with no record of injury (n = 185), C) horses that were between the age of 5–9 that never started in a race (n = 92) and D) random sampling of Thoroughbreds regardless of racing history or age (n = 279). Age range and gender distribution of each cohort are reported in Table 1. No first‐degree relatives were included in this study. All of these samples (with the exception of nine samples in cohort D) had genetic profiles on file and sample identity was confirmed by matching genetic profiles as described above.

### Genotyping and statistical tests

DNA was amplified for the *PLOD1* c.2032G>A variant using the commercially available assay at the VGL. To ensure accurate genotyping, assays were run with three positive controls (one for each genotype), and one negative control (water, no DNA). Positive controls genotyped as expected and negative controls did not yield detectable PCR product.

Power calculations were performed using a Pearson’s Chi‐squared test with a Cohen’s W calculation and varying sample sizes and allele frequencies. With a sample size of 200, allele frequency of 0.0575, and a frequency of cases set at 8% power to detect association is 81.87%. Therefore, with a total sample set of 716 individuals, even with a low allele frequency, sufficient power to detect associations should be achieved. Allele and carrier frequencies were calculated for each cohort and for the combined data set. Pairwise comparisons between the catastrophic breakdown cohort and control cohorts were evaluated by Fisher’s Exact test. Statistical significance was evaluated at both an uncorrected value of <0.05 (P_raw_) and Bonferroni corrected value (0.05/6 comparisons) of 0.008 (P_corrected_). Additional comparisons were made between the racing population and the cohort of horses that did not start in a race as well as between the racing cohort and the random population.

## Results

The WFFS allele (*PLOD1* c.2032G>A) was detected in all cohorts at a low frequency ranging from 0.5 to 1.8% (Table 3). Among all samples, the allele frequency was 1.2% (Table 3). Of the 716 horses tested in this study, 17 were carriers for the deleterious allele and no horses homozygous for the variant allele were identified.

**Table 3:** Genotyping results for the Warmblood fragile foal mutation (*PLOD1* c.2031G>A) in case and control cohorts of Thoroughbred horses. Presented are sample size, genotypes, and allele and carrier frequency for each cohort and the total population under investigation

Cohorts	Sample Size	WFFS genotype			Allele frequency	Carrier frequency
		*N/N*	*N/WFFS*	*WFFS/WFFS*		
Case cohort: catastrophic breakdown	22	21	1	0	0.023	0.045
Control cohort A: same track and same season as cases	138	135	3	0	0.011	0.022
Control cohort B: horses >7 years old and raced during same season as cases	185	183	2	0	0.005	0.011
Control cohort C: nonracers	92	91	1	0	0.005	0.011
Control cohort D: random population	279	269	10	0	0.018	0.036
Combined data	716	699	17	0	0.012	0.024

Only one horse in 22 catastrophic breakdown cases carried the WFFS allele. In an attempt to control for other environmental effects, we first compared the carrier and allele frequencies of the case population to a cohort of horses racing or training at the same track, during the same season as the cases (Cohort A; n = 138). There were no statistically significant differences between these pairwise comparisons (P = 0.5 for both comparison of allele and carrier frequencies). In evaluating an older cohort of horses (Cohort B; n = 185) that raced during the same season as the cases, but at racetracks across the United States, the number of carriers was not higher compared with the controls (P = 0.3), as might be expected if heterozygous WFFS horses were more prone to injury. We also sought to determine if there was a higher allele or carrier frequency among horses that did not race (Cohort C), but no significant differences were detected when comparing nonracers (n = 92) to either the case cohort (P = 0.4 carrier frequency and 0.3 allele frequency) or the combined racing cohort (P = 1.00). Finally, to investigate possible sampling biases in control cohorts, we also compared the allele and carrier frequencies of the case and the racing cohorts with those of a random sample of Thoroughbreds with no age criterion and whose racing records were unknown to the investigators (Cohort D; n = 279). No significant differences were detected in these comparisons (P = 0.6 for both allele and carrier frequency comparisons between cases and Cohort D and P = 0.2 for the comparisons between all racing cohorts and Cohort D). All association tests performed did not achieve a statistically significant probability value for the Bonferroni corrected P_corrected_ cut‐off.

## Discussion

The objective of this study was to estimate the *PLOD1* c.2032G>A allele frequency in the Thoroughbred breed and to determine if this allele was associated with catastrophic injury. By examining 716 horses, we confirmed the frequency of the *PLOD1* c.2032G>A variant to be low in all cohorts that we screened. Furthermore, these data do not support the WFFS allele as a risk factor for catastrophic breakdown. Specifically, differences were not detected in the *PLOD1* c.2032G>A allele or carrier frequencies between any of the cohorts under investigation. While cases from a single racetrack were analysed, the low allele frequency in each of the cohorts and in the combined data set makes it highly unlikely that this *PLOD1* variant plays a role in catastrophic breakdown.

This PLOD1 variant has been shown to cause a condition in Warmblood breeds similar to what has been described in humans as Ehlers‐Danlos syndrome, with fragile skin and lax and hyperextensible joints. The original study identifying the PLOD1 c.2032G>A variant was described in a patent application that reported two clinically WFFS‐affected horses homozygous for the variant (A/A), eight carriers (G/A) reported as clinically normal and 149 horses homozygous for the “G” allele, also clinically normal (International patent number WO 2012/158711). A subsequent clinical case report identified a third affected foal who was also homozygous for the PLOD1 variant and was euthanised because of poor prognosis 15. In humans, over 40 mutations in PLOD1 have been reported to cause Ehlers‐Danlos syndrome, kyphoscoliotic type 1. Some human patients show features of joint laxity and skin fragility, similar to WFFS in horse, while others are characterised by these and/or other clinical symptoms including congenital muscle hypotonia, congenital or early onset kyphoscoliosis, and scleral fragility and rupture of the globe of the eye 22, 23, 24. It is unknown if additional mutations are present in horse *PLOD1* that result in similar phenotypes.

Seventeen of 716 horses studied were carriers of this variant and no horse in this study was homozygous for the mutation. Based on the overall allele frequency of 1.2%, the expected incidence of WFFS in the Thoroughbred would be 1–2 horses in 10,000 births. With approximately 20,000 foals registered annually in the US 25 this would correspond to four cases each year. No cases of WFFS have been reported in Thoroughbred horses. However, one Ehlers‐Danlos Like Syndrome Thoroughbred case was reported in 1984 26. Although there are multiple genetic causes of Ehlers‐Danlos Like Syndromes this particular case, being an older horse with symptoms not as severe as those described for the WFFS mutation, makes it unlikely that it represents the same disease reported in Warmblood breeds 15. Given that there are no reported cases that clinically resemble WFFS in the Thoroughbred, despite the presence of the allele in the breed, albeit at a low frequency, it is plausible, that homozygosity for *PLOD1* c.2032G>A may result in early embryonic loss. In support of this, a recent study reported the frequency of the WFFS allele in Warmblood horses in Brazil to be 5.5%, with no live homozygotes and no reported WFFS cases in the country 27. The frequency of the PLOD1 variant in Warmbloods from other countries has not been reported. In addition, if homozygosity causes early embryonic loss in these breeds is yet to be investigated. Therefore, more work is needed to estimate allele frequency across breeds and investigate the embryonic loss hypothesis in horse breeds that possess the *PLOD1* c.2032G>A allele.

Heritability and genome‐wide association studies provide evidence that genetics plays a role in bone remodelling that contribute to catastrophic injury 13, 14. The present study determined that the *PLOD1* c.2032G>A variant is not a contributor to catastrophic injuries in the racing Thoroughbred. Therefore, the precise genetic variants that contribute to variation in bone remodelling remain to be identified.

## Authors’ declaration of interests

Drs Carrie Finno and Rick Arthur have no competing interests. Dr Rebecca Bellone, Dr Cecilia Penedo, Natalia Ocampo, Vince Le and Shayne Hughes are all affiliated with the UC Davis Veterinary Genetics Laboratory, which offers genetic testing for horses and other species including testing for the warmblood fragile foal mutation described in this manuscript.

## Ethical animal research

Tissues from horses that died or were subjected to euthanasia were obtained through sample submission through the California Animal Health and Food Safety Laboratory System following the protocols of California State Racing Board. All other samples were obtained from the Veterinary Genetics Laboratory archive with the approval of The Jockey Club.

## Owner informed consent

Representatives of the California Horse Racing Board and The Jockey Club authorised the genetic analysis in this study.

## Sources of funding

The project was supported by the UC Davis Veterinary Genetics Laboratory.

## Authorship

R.R. Bellone, M.C.T. Penedo and C.J. Finno contributed to concept of study and study design. N.R. Ocampo, S.S. Hughes, V. Le and R. Arthur contributed to acquisition of the data. R.R. Bellone and M.C.T. Penedo contributed to data analysis and interpretation of data. All authors contributed to manuscript preparation and approval of the submitted version of the manuscript.
